# The Prevalence and Treatment Costs of Non-Melanoma Skin Cancer in Cluj-Napoca Maxillofacial Center

**DOI:** 10.3390/medicina59020220

**Published:** 2023-01-23

**Authors:** Cosmin Ioan Faur, Mădălina Anca Moldovan, Mădălina Văleanu, Horațiu Rotar, Laura Filip, Rareș Călin Roman

**Affiliations:** 1 Department of Maxillofacial Surgery and Radiology, Oral and Cranio-Maxillofacial Surgery, “Iuliu Hațieganu” University of Medicine and Pharmacy, 33 Moților Street, 400001 Cluj-Napoca, Romania; 2 Department of Medical Informatics and Biostatistics, “Iuliu Hatieganu” University of Medicine and Pharmacy, 6 Pasteur Street, 400012 Cluj-Napoca, Romania

**Keywords:** non-melanoma skin cancer, head and neck cancer, hospitalization costs

## Abstract

*Background and Objectives:* An increasing incidence of non-melanoma skin cancer (NMSC) is noted, as well as an increasing cost of the treatment, with NMSC becoming a public health problem. We aimed to investigate the prevalence and treatment costs of surgically treated NMSC from the Oral and Maxillofacial Surgery Department of Cluj-Napoca County Hospital. *Materials and Methods:* We retrospectively analyzed the clinical data and the charge data of hospitalization from the informatic system of Cluj-Napoca County Hospital. All patients benefited from standard surgical excision with the reconstruction of the post-excisional defect. A statistical analysis of the costs related to the patients’ features, period and conditions of hospitalization, materials, medication, and paraclinical investigations was performed. *Results:* Between 2015 and 2019, 133 patients with NMSC were addressed to our department, with basal cell carcinoma (BCC) being four-fold higher than squamous cell carcinoma (SCC). Most NMSC cases were diagnosed in stage I or II, and they benefited from local reconstruction. The treatment costs progressively increased in the last five years, reaching a total cost of EUR ~13.000 in 2019. The treatment cost per episode was higher for SCC compared to BCC, while the total cost of treatment in 5 years was higher for BCC. Low income, immunosuppression, comorbidities, flap reconstruction option, long-lasting surgery, and prolonged hospitalization were associated with an increased cost of the treatment. *Conclusion:* The prevalence and treatment cost of surgically treated NMSC of the head and neck region increased in the last five years, with high-cost drivers being related to patients and treatment options.

## 1. Introduction

Non-melanoma skin cancer (NMSC) is an important health problem due to increased incidence in the last years and the increased morbidity and costs of the treatment [1,2]. However, the real incidence of NMSC is unknown. The World Health Organization (WHO) estimates 2–3 million malignant skin tumors per year which are most likely under-reported, especially in light of a recent study that estimated 3.3 million people who suffered from 5.4 million NMSC cases, part of them presenting more than one skin tumor [3]. In addition, the GLOBOCAN project of the International Agency for Research on Cancer reported an incidence of 1,198,000 new cases of NMSC (excluding basal cell carcinoma) in all sites worldwide in 2020, which was four-fold higher than malignant melanoma (324.000 new cases) [1]. The incidence of NMSC in the European population varies depending on geographical area, histopathological type, and age [4]. The prevalence of head and neck NMSC reported in Northeastern Romania was 72.5% and in Poland was 90% [5,6].

Most histopathological variants of the NMSC are basal cell carcinoma (BCC) (75–80%) and squamous cell carcinoma (SCC) (20–25%), the remnant variants (1%) being Merkel cell carcinoma, sebaceous carcinoma, apocrine adenocarcinoma, and other rare tumors [7]. In Europe, BCC presents a high incidence in Western Europe, especially in the Netherlands (87.5/100,000 person-years), Switzerland, and Italy (70/100,000 person-years), and a low incidence in Central and Eastern Europe, with Croatia presenting the lowest incidence (33.6/100.000 person-years). In addition to BCC, SCC has a high incidence in Western Europe (Switzerland 28.9/100.000 person-years), followed by Northern Europe, and a low incidence in Central and Eastern Europe (Croatia 8.9/100,000 person-years) [1,2,3,4].

The risk factors for developing NMSC are fair skin, increased number of freckles, sunburns, sun exposure, radiation, and immunosuppression [4,5,6,7,8]. Owing to the UV-associated risk, Australia and New Zealand are the countries with the highest risk of developing NMSC [1]. In addition, aging is an independent risk factor for the occurrence of head and neck NMSC. The head and neck region is prone to developing NMSC, being a highly sun-exposed area [5,6].

BCC causes significant morbidity due to local destructive spread and high local recurrence risk but has low metastatic potential [9]. On the contrary, SCC has a high propensity for metastatic spread, and hence a poor prognosis. SCC mortality is usually associated with locoregional and distant metastases [10]. The 5-year survival rates of NMS depend on age, immunosuppression status, surgical margins, and tumor features being higher for BCC compared to SCC [11]. Overall, NMSC has a better prognostic than other skin cancers, such as malignant melanoma or Merkel carcinoma [12,13].

NMSC treatment can be conducted by standard surgical excision, Mohs micrographic surgery, or destructive methods, such as curettage, cautery, cryosurgery, photodynamic therapy, and the application of local drugs [14]. Surgical excision, which encompasses 85% of cases, reports a five-year cure rate of 99% for BCC and 97% for SCC [15]. The cost of cancer treatment is high, being an economic and clinical burden to the National Healthcare system, as well as an economic burden to individuals [13]. The NMSC diagnosis and treatment costs are more expensive than those of malignant melanoma, due to higher incidence, even though the cost of malignant melanoma is higher per person-year [16,17]. Moreover, the NMSC treatment costs increased in the last years more than other skin cancers, being four-fold more expensive than malignant melanoma treatment [15,16,17,18]. BCC has a higher total cost of treatment compared with SCC [19]. Generally, the costs of head and neck cancers are higher compared with other locations, and the patients tend to be poorer and to have accrued lower levels of education than patients with other cancers [13,15,20]. Moreover, head and neck NMSC treatment has a higher cost than other anatomical regions, with the highest cost in nose BCC and auricle SCC tumors [5,16,19]. The costs also vary in different geographical areas due to different incidence and treatment protocols [21].

NMSC is not usually reported to a cancer database, hence the incidence and cost of the treatment are difficult to assess. Moreover, there are a few studies that discuss the head and neck NMSC treatment costs, and from our knowledge, none are reported in Romania. We aim to evaluate the prevalence and the direct costs of surgical treatment of head and neck NMSC from the Maxillofacial Surgery Department of Cluj-Napoca County Hospital.

## 2. Materials and Methods

We conducted a retrospective study on patients who were addressed to the Oral and Maxillofacial Surgery Department of Cluj-Napoca County Hospital between the first of January 2015 to the 31st of December 2019. We enrolled the patients who were hospitalized for at least 24 h in our department and had a histopathological result of head and neck BCC or SCC and primary or recurrent tumors. All the patients were treated by surgical excision and reconstruction of the defect. We excluded the patients who had an inconclusive histopathologic result, melanoma, Merkel cell skin cancer, or other rare malignant skin tumors, as well as benign skin lesions and the patients that underwent oncological treatment. All the included patients were staged according to American Joint Committee on Cancer (eighth edition) [22,23]. The patients’ comorbidities were classified based on the American Society of Anesthesiologists (ASA) risk stratification to have an objective assessment of the general health status [24]. Not all the patients responded to education formation and economical status at hospital admission. This study was approved by the Ethics Committee of “Iuliu Hatieganu” University of Medicine and Pharmacy (AVZ 20/03.02.2022), and it is in accordance with the updated Declaration of Helsinki.

### 2.1. Data Collection and Cost Analysis

We reviewed the clinical data and the charge data of hospitalization from the informatic system of Cluj-Napoca County Hospital. The data extracted from the electronic medical records included the following: patients’ demographics and associated medical diagnoses; the results of histopathological examinations; tumor characteristics, such as site; clinical and pathologic stage; treatment modality; and the discharge bill. The histopathological subtypes were classified according to the major pattern present in the specimen, many of them (89%) having 2 or more histopathological variants in the same specimen. We categorized the treatment after standard surgical excision as per primam closure, skin graft, and local flap reconstruction. 

We analyzed only the direct costs of care related to the hospitalization for NMSC surgical treatment (hospitalization accommodation costs, material costs, medication costs, and paraclinical examinations costs), without assessment of the direct costs of follow-up or the indirect (e.g., productivity costs) or intangible costs (e.g., monetary value of health loss and reduces the quality of life) [21,25]. The costs were presented as total treatment costs for NMSC, BCC, and SCC tumors and as individual mean costs of the tumors’ treatment related to the number of patients or episodes of cancer. A patient is defined as a person that suffered from at least one malignant skin tumor in the time interval. An episode (of cancer) is defined as a malignant skin tumor of a patient treated in one setting appointment (hospitalization). Hence, the means of cost per episode and per patient were constructed.

The costs are reported in Euros, with an exchange medium rate calculated after the Romanian National Bank rank (e.g., 5-year medium exchange rate Euro-RON). In addition, the costs are adjusted to the inflation rate up to 2022, according to the Romanian National Bank.

### 2.2. Statistical Analysis

The SPSS 25.0 software (SPSS Inc, Chicago, IL, USA) was used for statistical analysis and data description. The level of statistical significance was set at α =0.05. Mean ± standard deviation was used to describe normally distributed continuous quantitative data and absolute and relative frequencies (%) were used for qualitative data.

The comparison of the means was performed by the Student t-test for normally distributed data of two independent groups. The non-parametric Mann–Whitney and Kruskal–Wallis tests were used to compare the means of two independent groups with non-normal distribution. The Kruskal–Wallis test was also used for the comparison between more than two groups with non-normal distribution, and then the Tukey HSD test was used for post hoc analysis. Chi-Square or Fisher Exact tests were used to compare qualitative variables. Univariate regression analysis was used to estimate costs.

## 3. Results

### 3.1. Demographical and Epidemiological Consideration of the Population

**Figure 1:**
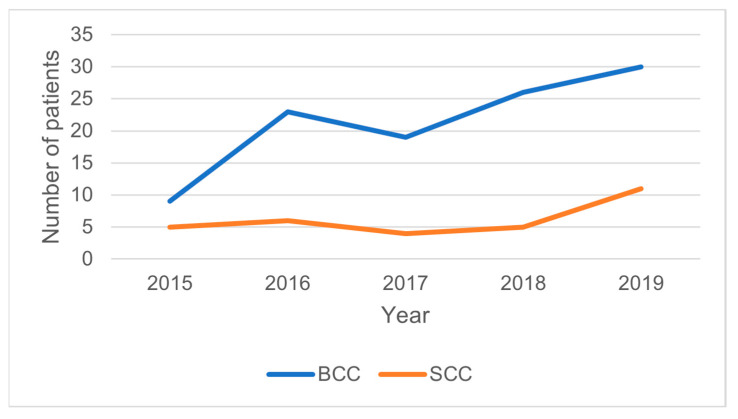
Year distribution of the skin cancer patients.

From a total of 195 patients who were addressed for skin tumors to our service between 2015 and 2019, 144 patients presented malignant tumors, including 133 (92%) BCC and SCC patients (Figure 1). This study enrolled 102 (76%) BCC patients, 27 (20%) SCC patients, and 4 (4%) patients with both skin cancers, which presented 152 episodes of cancer. Most of the BCC tumors were nodular (66 tumors), infiltrative (33 tumors), or basosquamous (13 tumors) differentiated, the rest (8 tumors) being less frequent histopathological BCC variants. SCC grading revealed 11 well-differentiated, 18 intermediate-differentiated, and 3 poorly differentiated tumors, with only 5 of the SCC tumors (15%) being non-keratinizing lesions. Sixteen patients had two NMSCs with different localizations, excised during the 5-year period (eleven BCC patients, one SCC, and four patients had one BCC and one SCC). One 74-year-old female patient presented four hospitalizations between 2018 and 2019 for BCC tumors located in different head and neck regions.

The mean ± standard deviation age of the NMSC patients was 67 ± 13 years, being slightly increased for SCC subpopulations (69 ± 15 years) compared to SCC ones (67 ± 13 years). The maximum prevalence of NMSC episodes was in the seventh decade of life, as can be seen in Table 1. The male-to-female ratio was 1.3:1 and most of the patients (60.53%) had an urban place of residence. In addition, most of the patients (61 subjects) that responded to the admission questionnaire did have any education following high school, and only 32 patients had university studies. In addition, many of these patients had low-income economic status.

**Figure 2:**
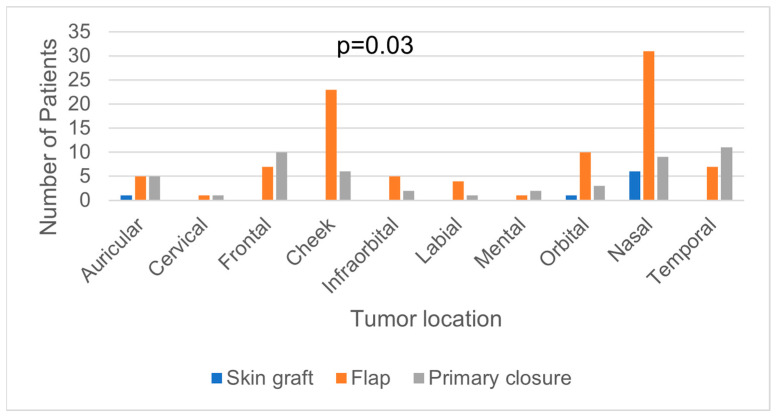
Correlation between tumor location and type of reconstruction used to repair the surgical defect.

We found 148 primary tumor excisions of NMSC (117 BCC and 31 SCC) and 4 recurrence tumor excisions (3 BCC and 1 SCC). The tumor distribution in the head and neck regions is seen in Table 1. The nasal and cheek regions were highly involved by NMSC tumors. The skin malignant tumors were diagnosed frequently in stage I (97 tumors) or II (31 tumors) and were frequently managed by radical excision (146 tumors) with flap reconstruction (84 cases) or primary closure (50 cases) (Table 1). The surgical defects located in nasal, cheek, and orbital regions often required flap reconstructions, and the ones located in frontal and temporal areas had more primary closures than flap reconstructions (*p* < 0.05) (Figure 2).

The surgical excisions and reconstructions last less than 1 h or between 1 and 2 h for most of the tumors (Table 1). The patients were hospitalized between 1 (61 patients) and 10 days (1 stage IV patient), with a median (Q1, Q3) of 2 (1;3) days of hospital staying. A total of 342 days (264 BCC and 78 SCC) were needed for the head and neck skin cancer treatment in the 5 years.

**Figure 3:**
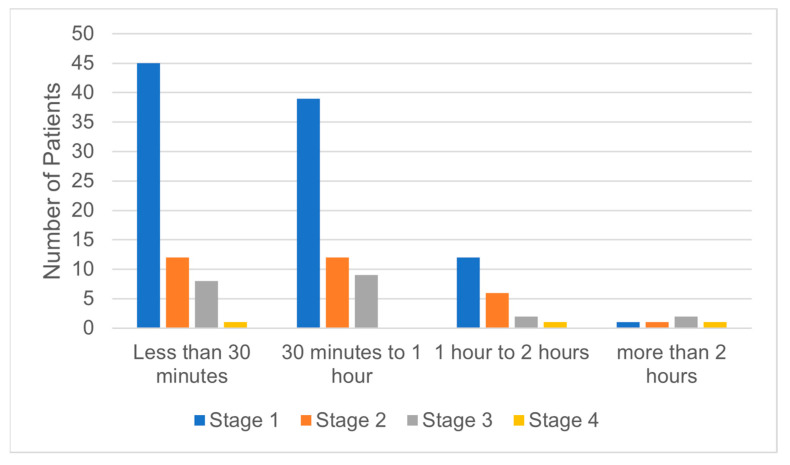
Correlation between tumor stage and time required to perform surgery.

**Figure 4:**
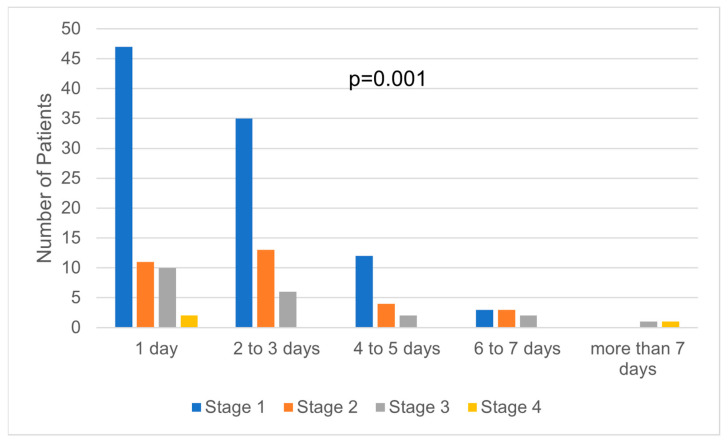
Correlation between tumor stage and days of hospitalization.

**Figure 5:**
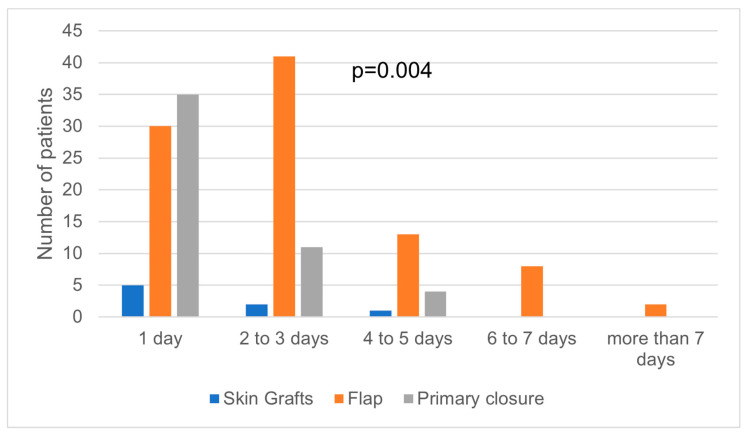
Correlation between days of hospitalization and type of reconstruction.

**Figure 6:**
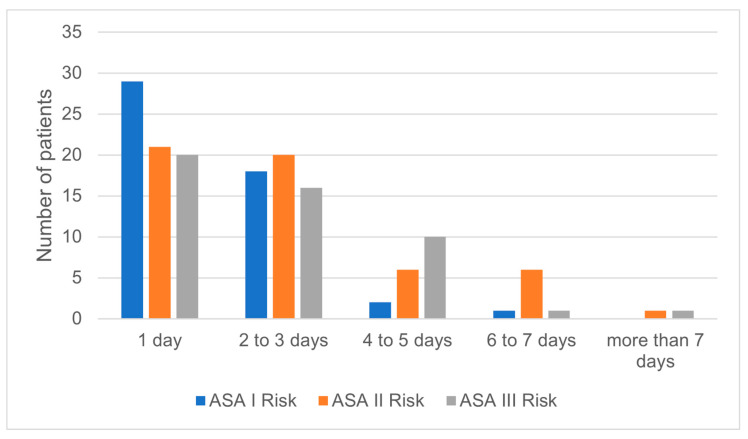
Correlation between days of hospitalization and ASA risk.

Even if the first stage of NMSC cases were frequently surgically treated in less than one hour, there was no statistical significance regarding the correlation between the tumor stage and the time required by surgery to be performed (Figure 3). However, there was a statistically significant correlation between the tumor stage and the hospitalization days. Stages I and II were mostly treated within one to three days of hospitalization (Figure 4). In addition, the days of hospitalization were statistically correlated with the type of reconstruction, with primary closure and skin grafts requiring fewer hospitalization days than local flap reconstruction (Figure 5). On the contrary, the hospitalization days were not statistically correlated with ASA risk, even though most ASA risk I patients required one to three days of hospitalization (Figure 6).

### 3.2. The Cost of NMSC Treatment

**Figure 7:**
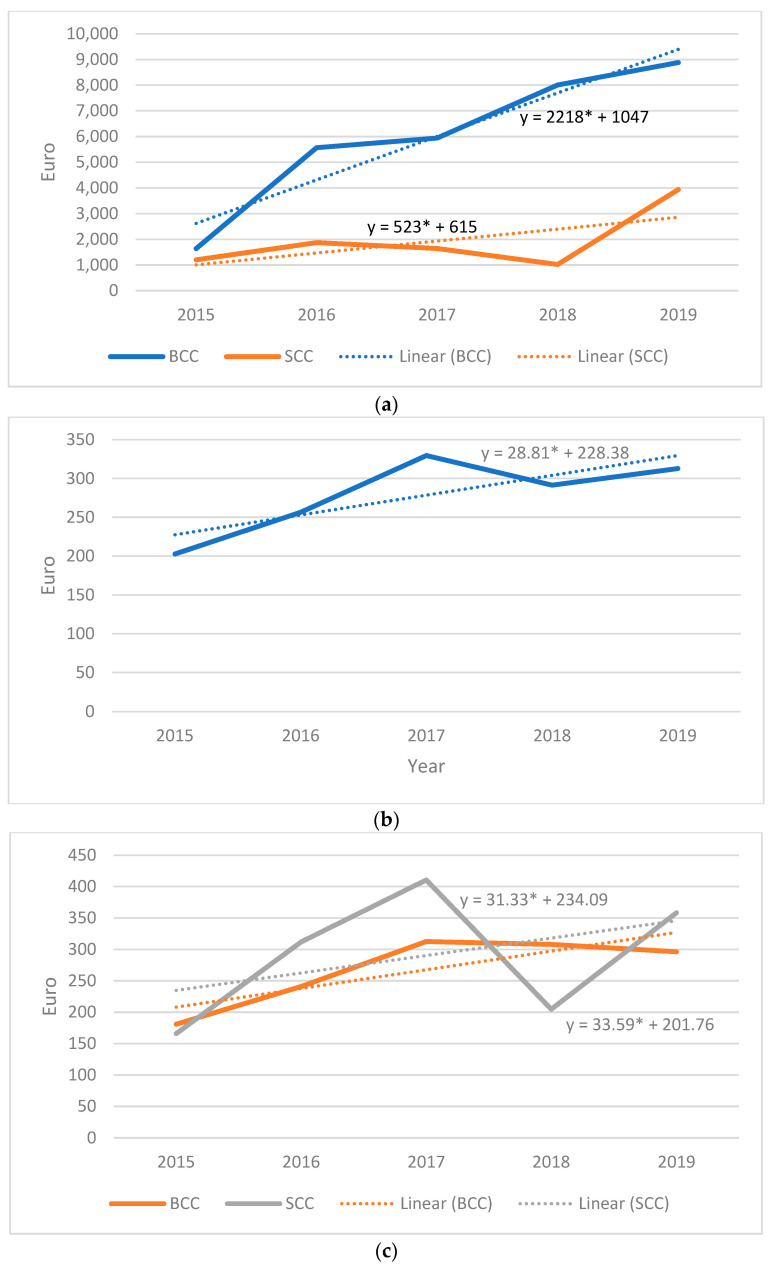
Direct treatment costs of NMSC: (**a**) total costs/5 years for SCC and BCC; (**b**) the increasing NMSC treatment costs/episode during 5 years of analysis; (**c**) the increasing BCC and SCC treatment costs/episode during 5 years of analysis.

The direct costs of NMSC in 5 years of treatment were approximately EUR 45.000, with an average cost/patient and cost/episode of EUR 336 and EUR 294, respectively. The costs of treatment/episode had a statistically significant increase (*p* = 0.03) from EUR 228 in 2015 to EUR 352 in 2019, with a mean increase of approximately 28 EUR/year (Figure 7). The total treatment cost/5 years was more expensive (approximately EUR 34.000) for BCC than SCC (approximately EUR 11.300). However, the average treatment cost/patient was higher for SCC (EUR 352) than for BCC (EUR 316). The most expensive treatment (EUR 2064) was applied to a temporal BCC patient that was hospitalized for 10 days in 2017. 

Additional to the costs of the surgery and the accommodation, the total direct costs of treatment included EUR 2774 for the drugs administrated during the hospitalization, EUR 2384 for the supplies, and EUR 5670 for the paraclinical examinations for the 5 years of analysis. However, the costs of the surgery and the accommodation cannot be differentiated and individually assessed due to the lack of data illustrated in the informatic system of the hospital. The details of the treatment costs are seen in Table 2. Except for the medication, the medium cost of the supplies and paraclinical examination was higher for SCC in comparison with BCC.

The average cost per day of treatment for NMSC was EUR 131, with SCC being more expensive to treat (EUR 138) in comparison with BCC (EUR 127). However, the cost increased with the days of hospitalization, being 9-fold higher for more than 7 days of hospital staying than for 1 day of hospitalization.

### 3.3. Cost of NMSC Treatment Related to the Demographical and Clinical Features

Starting from the third decade of life, the treatment cost of NMSC statistically significantly increased (*p* = 0.04) by approximately 10% to 25% per decade (Table 1). The male patients and the subjects that had a rural place of living presented higher costs of treatment, however, without any statistical significance. Moreover, the patients that underwent technical schools and subjects with lower income presented higher costs of treatment, with the economic status classification being the only statistically significant result (*p* = 0.004). 

Immunosuppression was a general status-related factor that influenced the treatment costs, with immunosuppressed patients presenting a statistically significant higher cost of treatment compared with the non-immunosuppressed subjects (Table 1). ASA risk classification also statistically increased the cost of NMSC, with the third class presenting a 39% higher cost compared to the first class.

The orbital region presented the highest cost of NMSC treatment. In addition to tumor location, the tumor stage did not present statistical significance, even if stage IV had approximately 4-fold increased treatment costs compared to stage I.

Re-excision costs were 1.57-fold higher than radical treatment of a primary tumor, without any statistical significance. In addition, standard excision surgery associated with flap reconstruction or surgery that lasts more than two hours presented the highest treatment cost (*p* < 0.001).

## 4. Discussion

The prevalence of NMSC tumors of the head and neck region in our study (approximately 90% of the malignant skin tumors) was higher than that reported in northeastern Romania, however, the BCC to SCC ratio (4:1) was in accordance with the literature [1,5,6]. In addition, we had similar results regarding the age-related risk for NMSC development, with approximately 80% of the episodes of cancer in our research occurring in subjects older than 60 years, with a slightly increased age of SCC subjects [21,26]. We found that the nose was the most frequent tumor site for NMSC lesion development.

We identified increasing costs of NMSC treatment per year in the last 5 years, which could be attributed to the increasing number of patients and to the treatment costs of cancer episodes, expected to be much higher in the next years as the costs were constantly raising by 28 EUR/year. The increasing costs of the treatment rely on the new technologies and better quality of life offered by the new therapies [27]. However, our findings underestimate the real total costs of NMSC treatment, which has a complex calculation [20,26]. The direct cost of treatment represents approximately 72% of the total cost related to NMSC treatment, without including the oncological treatment, follow-up, patients’ loss of income, quality-of-life changes, and economic reintegration [21,25] 

We included only patients that were addressed to a Maxillofacial hospital, without subjects that were treated by other specialists, such as Plastic Surgery or Dermatology physicians, where the treatment options and the associated costs may be different from our standard surgical excision [15,21,26]. In addition, this research was limited to the hospitalized patients and charge data, aiming to identify the economic burden of the healthcare system and to observe the cost distribution patterns and high-cost drivers. However, the costs of hospitalized patients are higher than ambulatory-treated patients [15]. Still, the cost of NMSC treatment in Romania is cheaper than in other countries, such as Australia or other Northern or Western European countries [21,26].

Different from other studies, we could not find an association between the place of living or sex and NMSC increased cost. In the research of Tran et al. and Doran et al., urban place of residence or female patients presented higher costs of NMSC treatment compared with rural place of living and male patients [19,21]. Elderly patients presented a higher cost of treatment compared to young adults due to various arguments. Firstly, the prevalence of NMSC in senior people is higher compared to young adults [21,26]. Secondly, elderly people require special medical attention due to the increased prevalence of comorbidities, quantified in our study by the ASA risk. The patients classified as high ASA risk had a significantly higher cost compared to the low-risk subjects [20]. However, our results did not indicate a statistical correlation between the increased number of hospitalization and advanced ASA risk. In addition, immunosuppressed patients had a higher cost of treatment compared with non-immunosuppressed ones, with five out of the six immunosuppressed patients included in this study being aged more than 60 years old.

The increased costs of NMSC treatment for technical school and low-income patients were consistent with other research. The subjects that are exposed more to professional emissions and have low income are prone to developing NMSC cancers, and the treatment costs are higher compared with patients of other education and economic status [6,20]. However, we found a statistical significance of increasing costs related only to educational formation.

The head and neck NMSC treatment is more expensive than other anatomical regions, such as the thorax and limbs, but it is lower compared with head and neck mucosal malignancies (e.g., SCC of oral cavity or oropharynx) [19,20]. However, 19% of head and neck cancers are located on the skin [25]. Particularly, the orbital region had the highest cost of treatment per episode, which may be due to the difficulty of local reconstruction of this anatomical area [28,29]. These results are different from other research, where nasal and auricle NMSC had the highest treatment costs [5,16,19]. In our research, the nasal region presented an average cost of treatment per episode lower than the orbital region, but the total cost of treatment was the highest compared with other anatomical regions, due to the highest number of NMSC episodes. 

Although the treatment cost of BCC per 5 years was higher compared to SCC, the BCC treatment cost per episode was cheaper than SCC, similar to other research [19,26]. These findings may be explained by the increased rates of BCC tumors and the more radical treatment required by SCC tumors, as well as the more paraclinical examinations needed perioperatively by SCC tumors to evaluate the locoregional or distant metastasis [17,30,31].

Different from the study by Chen et al. that observed a significant increase in treatment costs in the advanced stages of the disease, we observed an approximately 4-fold increase in the treatment costs of stage IV compared with stage I, but our results had no statistical significance [15]. However, the costs may be related to the more complex surgical procedure and to the more careful postoperative medical attention in advanced stages compared to early stages [32].

The local flap was approximately 60% more expensive than the skin graft. The result of this type of reconstruction is more aesthetic compared to the skin graft due to its similar color and texture to the adjacent facial tissues [33]. Both variants of reconstruction are used for tumors of increased dimensions, where the primary closure is not possible. However, the local flaps require an increased procedure time compared to other types of reconstruction, with the time needed to perform the surgery also being a factor that increased the costs [25]. A procedure that lasts longer than 2 h has more than double the costs compared with surgeries that can be performed faster (1 h). In addition, the local flap required more days of hospitalization than the primary closure or skin graft.

In our study, the high length of hospitalization was associated with higher costs of treatment. This finding may rely on the general health status of the patient or on the complex surgical procedure that requires many days of medical attention [31]. However, we found a statistical correlation between the hospital stay and the reconstruction type and tumor stage, without ASA risk. The median length the hospitalization for NMSC patients was 2 days, indicating that the surgical treatment of NMSC can be performed as a fast-track surgery, especially for patients with low ASA risk and early stages of cancer. 

We enrolled a limited number of patients, which may influence the statistical analysis. Due to COVID-19 lockdowns, the number of patients that were addressed to Oral and Maxillofacial Surgery for head and neck skin tumors was reduced, hence, we limited the inclusion criteria to the 31st of December 2019 [34]. However, the majority of included patients were clustered in stage I or II, similar to other research [35].

Another limitation of our study was the lack of capacity to particularly assess the cost of the surgical procedure due to the lack of specific data in our hospital’s informatic system.

## 5. Conclusions

Non-melanoma skin cancers are an important health problem due to increasing prevalence and treatment costs in the last years. The factors that can increase the costs of the treatment are related to the patient, such as age, low income, immunosuppression, and comorbidities, and to the procedure, such as flap reconstruction option, long-lasting surgery, and prolonged hospitalization.

## Figures and Tables

**Table 1:** Non-melanoma skin cancer epidemiology and treatment costs.

	Malignant Skin Cancer Episodes				NMSC Treatment Cost Per Episode	
	NMSC *n* = episodes (*n*%)	BCC *n* = episodes (*n*%)	SCC *n* = episodes (*n*%)	*p* value (BCC vs. SCC among subsets) *	Mean ± standard deviation (Euro) **	*p* Value (costs comparison among subsets) ***
**Age decade distribution**						
20–30	2 (1.31%)	1 (0.83%)	1 (3.13%)	0.31	110 ± 9	0.04
30–40	5 (3.27%)	4 (3.33%)	1 (3.13%)		177 ± 67	
40–50	18 (11.76%)	15 (12.5%)	3 (9.38%)		205 ± 90	
50–60	5 (3.27%)	4 (3.33%)	1 (3.13%)		226 ± 42	
60–70	37 (24.18%)	33 (27.5%)	4 (12.5%)		248 ± 150	
70–80	54 (35.29%)	41 (34.17%)	13 (40.63%)		281 ± 224	
80–90	30 (19.61%)	22 (18.33%)	8 (25%)		352 ± 210	
90–100	1 (0.65%)	-	1 (3.13%)		-	
**Sex**						
Female	64 (42.1%)	49 (40.83%)	15 (46.85%)	0.53	323 ± 271	0.17
Male	88 (57.9%)	71 (59.17%)	17 (53.13%)		271 ± 245	
**Place of living**						
Rural	60 (39.47%)	45 (37.5%)	15 (46.88%)	0.33	331 ± 327	0.44
Urban	92 (60.53%)	75 (62.5%)	17 (53.13%)		266 ± 190	
**Educational formation**						
Elementary school	5 (3.76%)	2 (1.87%)	3 (9.68%)	0.13	331 ± 329	0.16
Medium school	24 (18.05%)	21 (19.63%)	3 (9.68%)		242 ± 159	
Technical school	9 (6.77%)	8 (7.48%)	1 (3.23%)		523 ± 622	
High School	19 (14.29%)	16 (14.95%)	3 (9.68%)		258 ± 293	
College	15 (11.28%)	11 (10.28%)	4 (12.9%)		214 ± 163	
**Economic status**						
Low income	42 (27.45%)	23 (19.17%)	10 (31.25%)	0.66	300 ± 331	<0.01
Medium income	26 (16.99%)	47 (39.16%)	3 (9.38%)		283 ± 282	
Upper income	4 (2.61%)	3 (2.5%)	1 (3.13%)		103 ± 4	
**Immunosuppression**						
Yes	6 (3.95%)	3 (2.5%)	3 (9.38%)	0.07	419 ± 212	0.04
No	146(96.05%)	117 (97.5%)	29 (90.63%)		277 ± 213	
**ASA risk classification**						
I	50 (32.89%)	40 (33.33%)	10 (31.25%)	0.91	242 ± 219	0.03
II	54 (35.53%)	42 (35%)	12 (37.5%)		333 ± 106	
III	48 (31.58%)	38 (31.67%)	10 (31.25%)		339 ± 142	
**Tumor’s location (head and neck regions)**						
Auricular	11 (7.24%)	6 (5%)	5 (15.63%)	0.14	214 ± 113	0.14
Cervical	2 (1.32%)	1 (0.83%)	1 (3.13%)		215 ± 49	
Frontal	17 (11.18%)	11 (9.17%)	6 (18.75%)		332 ± 303	
Cheek	29 (19.08%)	24 (20%)	5 (15.63%)		329 ± 225	
Infraorbital	7 (4.61%)	6 (5%)	1 (3.13%)		203 ± 61	
Labial	5 (3.29%)	4 (3.33%)	1 (3.13%)		376 ± 225	
Mental	3 (1.97%)	3 (2.5%)	-		143 ± 33	
Orbital	14 (9.21%)	13 (10.83%)	1 (3.13%)		421 ± 340	
Nasal	46 (30.26%)	40 (33.33%)	6 (18.75%)		256 ± 154	
Temporal	18 (11.84%)	12 (10%)	6 (18.75%)		301 ± 456	
**Stage of the disease**						
I	97 (63.82%)	83 (69.17%)	14 (43.75%)	0.002	253 ± 151	0.65
II	31 (20.39%)	25 (20.83%)	6 (18.75%)		307 ± 184	
III	21 (13.82%)	11 (9.17%)	10 (31.25%)		371 ± 446	
IV	3 (1.97%)	1 (0.83%)	2 (6.25%)		974 ± 715	
**Type of reconstruction**						
Primary closure	50 (32.68%)	37 (30.83%)	13 (40.63%)	0.52	198 ± 116	<0.01
Skin graft	8 (5.23%)	6 (5%)	2 (6.25%)		220 ± 134	
Local flap	94 (61.44%)	77 (64.17%)	17 (53.13%)		352 ± 315	
**Time needed for surgical procedure**						
<1 h	66 (43.42%)	53 (44.16%)	13 (40.62%)	-	257 ± 177	<0.01
1–2 h	66 (43.42%)	52 (43.33%)	14 (43.75%)		340 ± 155	
>2 h	20 (13.16%)	15 (12.5%)	5 (15.62%)		647 ± 581	
**Days of hospitalization**						
1	69 (45.39%)	56 (46.66%)	13 (40.62%)	0.53	139 ± 31	<0.01
2	36 (23,68%)	31 (25.83%)	5 (15.62%)		281 ± 190	
3	18 (11,84%)	13 (10.83)	5 (15.62%)		357 ± 46	
4	14 (9,21%)	9 (7,5%)	5 (15.62%)		475 ± 58	
5	4 (2,63%)	3 (2.5%)	1 (3.12%)		596 ± 29	
6	5 (3,28%)	4 (3.33%)	1 (3.12%)		627 ± 70	
7>	6 (3,94%)	4 (3,33%)	2 (6.25%)		1177 ± 526	

* Statistical analysis of episode comparison between BCC and SCC subsets; ** average treatment costs of NMSC related to the cancer episodes; *** statistical analysis of NMSC cost between different subsets.

**Table 2:** Averages of direct cost details of head and neck skin cancers for five years of treatment related to patient and episode of cancer.

Costs (EUR)	NMSC	BCC	SCC
**Total direct costs**			
Average cost per patient	336	317	252
Average cost per episode	294	282	341
**Drug costs**			
Average cost per patient	20	22	13
Average cost per episode	18	19	12
**Paraclinical examination costs**			
Average cost per patient	42	38	49
Average cost per episode	37	33	48
**Supply costs**			
Average cost per patient	18	17	17
Average cost per episode	15	15	17

## Data Availability

Not applicable.
